# Role of viral evolutionary rate in HIV-1 disease progression in a linked cohort

**DOI:** 10.1186/1742-4690-2-41

**Published:** 2005-06-29

**Authors:** Meriet Mikhail, Bin Wang, Philippe Lemey, Brenda Beckthold, Anne-Mieke Vandamme, M John Gill, Nitin K Saksena

**Affiliations:** 1 Retroviral Genetics Laboratory, Center for Virus Research, Westmead Millennium Institute, Westmead Hospital, The University of Sydney, Westmead NSW 2145. Sydney, Australia; 2 Department of Clinical and Epidemiological Virology, Rega Institute, Minderbroedersstraat 10, B-3000 Leuven, Belgium; 3 Department of Medicine, University of Calgary, 3330 Hospital Drive NW Calgary, Albert, T2N 4N1, Canada

## Abstract

**Background:**

The actual relationship between viral variability and HIV disease progression and/or non-progression can only be extrapolated through epidemiologically-linked HIV-infected cohorts. The rarity of such cohorts accents their existence as invaluable human models for a clear understanding of molecular factors that may contribute to the various rates of HIV disease. We present here a cohort of three patients with the source termed donor A – a non-progressor and two recipients called B and C. Both recipients gradually progressed to HIV disease and patient C has died of AIDS recently. By conducting 15 near full-length genome (8.7 kb) analysis from longitudinally derived patient PBMC samples enabled us to investigate the extent of molecular factors, which govern HIV disease progression.

**Results:**

Four time points were successfully amplified for patient A, 4 for patient B and 7 from patient C. Using phylogenetic analysis our data confirms the epidemiological-linkage and transmission of HIV-1 from a non-progressor to two recipients. Following transmission the two recipients gradually progressed to AIDS and one died of AIDS. Viral divergence, selective pressures, recombination, and evolutionary rates of HIV-1 in each member of the cohort were investigated over time. Genetic recombination and selective pressure was evident in the entire cohort. However, there was a striking correlation between evolutionary rate and disease progression.

**Conclusion:**

Non-progressing individuals have the potential to transmit pathogenic variants, which in other host can lead to faster HIV disease progression. This was evident from our study and the accelerated disease progression in the recipient members of he cohort correlated with faster evolutionary rate of HIV-1, which is a unique aspect of this study.

## Background

The rate of HIV disease progression varies greatly among infected individuals, which is defined invariably by increasing plasma viral loads and concomitant decline in the CD4^+ ^T cell counts. A small but rare subset of chronically-infected individuals comprising <0.8% of total HIV infected population appear to maintain high and stable CD4^+ ^and CD8^+ ^T cell counts, low to undetectable plasma viral loads for >10 years in the absence of antiretroviral therapy [1,2]. In addition, some of these non-progressing individuals harbor <10 copies of proviral DNA/ml blood, show strong immune responses [2,3] and a high secretion of CD8 antiviral factor(s) (CAF) [3,4]. Additionally, in rare cases there is a complete absence of viral evolution over time [5].

HIV disease is a complex interplay of both host and viral factors [6-10], but it has been difficult to derive a consensus on these factor(s) that contribute to disease progression and / or non-progression. In many cases, evidence suggests that viral gene defects contribute to non-progression of HIV disease [6,11-14], yet these molecular changes remain elusive due to the extensive inter-strain variation of HIV-1, which can be investigated using epidemiologically-linked cohorts. The rarity of such cohorts accents their existence as invaluable models for understanding how various host and viral factors govern HIV pathogenesis. For such purposes, we describe detailed molecular analyses of one such cohort comprising of 3 HIV-infected individuals (a non-progressing donor-A and two recipients B and C) whose epidemiological linkage was confirmed through phylogenetic analyses [15]. The donor A likely acquired HIV in 1982, and has remained healthy maintaining non-progressive status with high CD4^+ ^and CD8^+ ^T cell counts and with <7000 HIV-1 copies/ml of plasma. The two recipients were infected in autumn 1983 (recipient B) and in summer of 1983 (recipient C) respectively.

With the help of detailed full-length HIV-1 genome analysis over time from all cohort members, we investigated viral evolution, divergence, recombination and selective forces in contributing to HIV disease development in the two recipients as opposed to the non-progressive donor.

## Results

### Sequencing of near full-length genomes

Successful amplification of near full-length HIV-1 genomes was achieved from a total of 15 PBMC patient samples collected between 1992 to 2000 from all 3 cohort members A, B and C. Epidemiological-linkage was confirmed by maximum likelihood phylogenetic analysis which was subsequently used for further intra patient evolutionary analysis as discussed previously in Mikhail *et al*., 2005 [15].

### Phylogenetic clustering of cohort members: evidence of HIV transmission via blood transfusion

Within the HIV-1 subtype B phylogenetic tree, the cohort clearly constitutes a single cluster, supported by high bootstrap values as posterior probabilities. Interestingly, the donor A lineage appears to be the out group for the two recipients and it was noted that recipient C revealed one long-branch segregating earlier time points from samples obtained from 1997 till 2000 [15]. As this is in correlation to clinical patient profile, one can deduce that the emergence of host-induced viral variation and hence viral evolution at recent time points occurred in concert with the rapidly progressing status of AIDS patient C. This pattern was also evident through analyses obtained from all the individual genes (data not shown).

**Figure 2:**
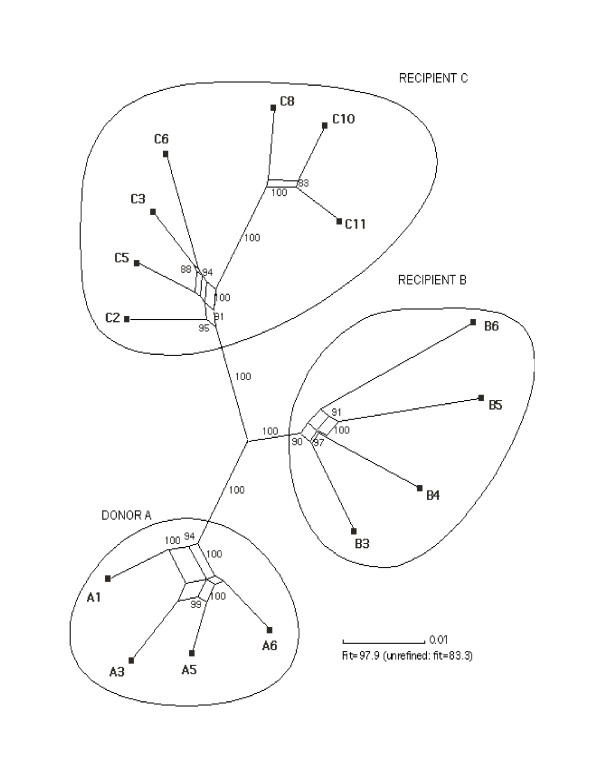
Split graph of the cohort reconstructed using the Kimura-2-parameter corrected distances. The splits were refined since this significantly improved the fit. Bootstrap values are indicated on the edges and were performed using the Neighbor-Joining method on 1000 replicates (previously published in Mikhail *et al*., 2005). Bayesian trees were reconstructed in mrBayes v2.01. Network analysis was performed in Splitstree v 1.0.1, 2.4; Huson 1998).

Overall, patient-derived virus sequences obtained from corresponding longitudinal samples showed tight clustering within patients, well supported by bootstrap values and posterior probabilities. To analyze within patient evolutionary patterns, a splitstree, allowing the representation of conflicting phylogenetic signal, was reconstructed for all the cohort sequences (Figure 2). In the splitstree the evolutionary patterns within each patient are blurred by discordant relationships indicated by the reticulate pattern of evolution. This pattern of phylogenetic discordance suggests the presence of recombination and/or adaptive evolution, which is acting as a major evolutionary force on the patient's viral variants over time *in vivo*. Recombination produces networks of sequences rather than strictly bifurcating evolutionary trees. Depicted by the Splitstree program, a tree topology typical of recombination or conflicting phylogenetic signals in the data contains parallel edges between sequences.

### Recombination analysis

To further delineate the cause of net like pattern seen at the nodes of the splits tree and to determine whether recombination has shaped the evolution of viral sequences, the Informative Sites Tests (IST) together with the Homoplasy test was conducted to test whether the null hypothesis of pure clonal evolution can be significantly rejected [16,17]. In addition, we also attempted to quantify the contribution of recombination to the viral genetic diversity using the Informative Site Index and the Homoplasy Ratio (HR) (Table 1). For the complete genomes, both indices are in the same order of magnitude of 0.3 indicating the presence of recombination. However, for the major genes, the P values still indicate the hallmark of recombination, but the recombination indices become slightly varied and are no longer comparable between the two tests. If this recombination signal is also the cause of reticulate evolution within each patient, then recombination was equally evident in both the donor and recipients (Figure 2). Therefore, even though recombination appears to be an inherent property in this cluster, its exact biological association with progression and non-progression of HIV disease in this cohort is only partially clear, and the possible role of selection pressures on disease progression is needed to be investigated.

**Table 1:** Results of the Homoplasy Test and the Informative Sites Test

	Homoplasy Test		Informative Sites Test	
	P value	HR	P value	ISI
complete genome	P < 0.001	0.254	P < 0.001	0.34
*gag*	P < 0.017	0.565	P < 0.098	0.38
*pol*	P < 0.015	0.299	P < 0.007	0.41
*env*	P < 0.043	0.152	P < 0.002	0.42

### Selective pressure and evolutionary rate analysis

**Figure 3:**
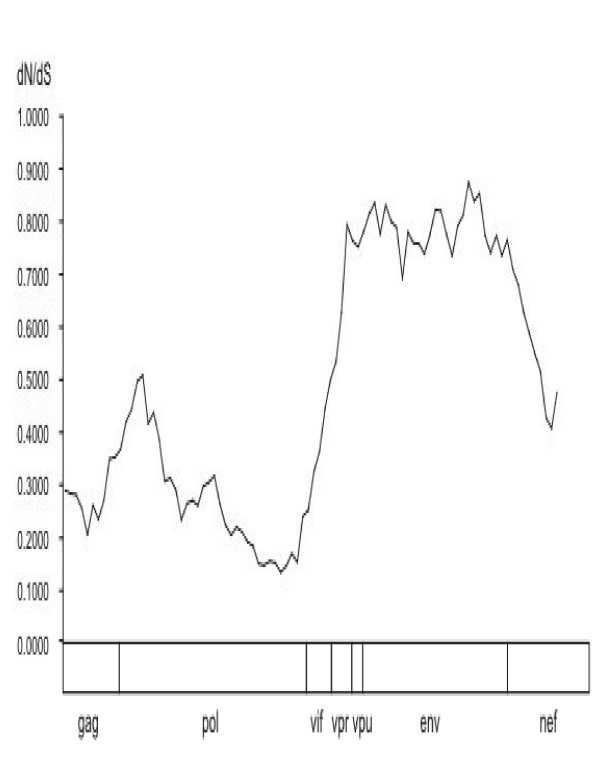
Non-synonymous : synonymous base rate ratio across the complete genome as estimated under a codon substitution model (MO) in a sliding window fashion with a step size of 81 bp and a window size of 801 bp, indicating the highest ratios within the *en*v gene, followed by the *pol, gag *and nef genes, respectively.

To investigate the selective pressure exerted on the virus in the cohort members, a non-synonymous/synonymous substitution rate ratio scan was performed on the complete genomes using a maximum likelihood estimation procedure (Figure 3). The average *d*N/*d*S ratio shows considerable variation across the genome, with the highest ratios in the *en*v gene, intermediate values in the accessory genes and lower values in the *pol *gene, with fairly low values for the *gag *gene. A similar analysis using complete genomes, representative for the HIV-1 diversity group M found from the Los Alamos HIV Database, also resulted in a similar plot, confirming previous reported results [9,17,18]. With the methods at hand, we can quantify the selective pressure across the genome for the complete cohort but it is not possible to document differences in selective pressure between cohort members due to parameter constraints of the mathematical models used. Thus, although over time analyses do demonstrate that differential selective pressure is clearly present in this cohort, its clear relationship with disease progression cannot be unraveled due to the possible contributing role of recombination. And since selection can result in heterogeneous rates along sequences, conflicting phylogenetic signal in this cohort might also have arisen from selection in addition to recombination. This is further confirmed by the correlation of the log likelihood estimates of the overall phylogenetic hypothesis plotted against the *dN/dS *ratios obtained by the scanning window approach (data not shown).

**Figure 4:**
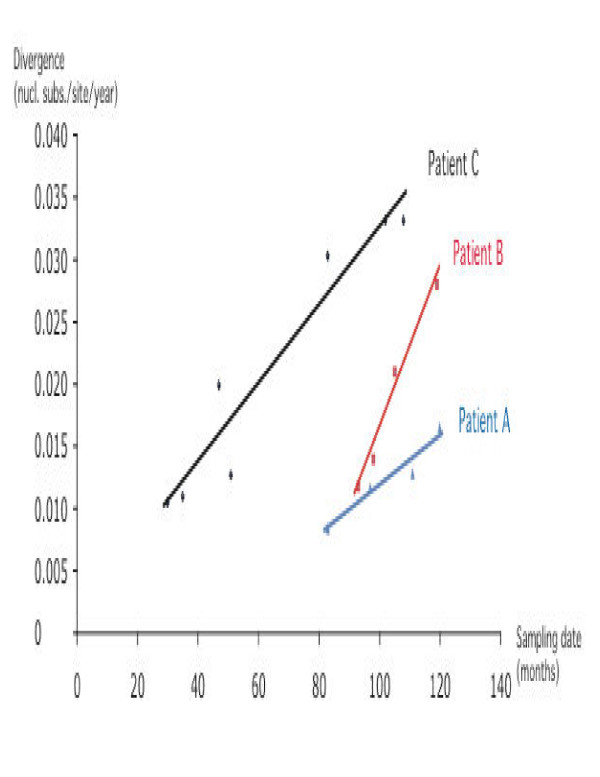
Linear regression plot for root to tip divergence versus sampling date within each patient of the cohort. All regressions had an R^2 ^value above 0.92. This graph indicates the highest slope and thus evolutionary rate for recipient B, followed by recipient C and lowest evolutionary rate for non-progressing donor A.

To investigate differences in evolutionary rate between patients, molecular clock analysis was performed. Figure 4 shows the root-to-tip divergence in function of the sampling time. Linear regression estimates for the evolutionary rates were 2.38 × 10^-3 ^(7.33 × 10^-4^-3.87 × 10^-3^), 7.75 × 10^-3 ^(1.86 × l0^-3^-8.38 × 10^-3^) and 3.77 × 10^-3 ^(3.07 × 10^-3^-4.44 × 10^-3^) nucleotide substitutions/site/year for patient A, B and C, respectively (Figure 4). By incorporating a global molecular clock, constraining all branches with one single evolutionary rate, and local molecular clocks, accommodating for different rates among different branch sets, evolutionary rates were obtained by maximum likelihood under the tip-dated model. Table 2 shows that allowing for different rates among the patients provided a significantly better fit (P < 0.001) than the global clock model, illustrating that the evolutionary rates were significantly different for the three cohort members. It should be noted however that the non-clock model, allowing for a different rate for each branch in the phylogeny, still remained significantly better as determined by the likelihood ratio test. Estimates of the evolutionary rate show a slow evolution for patient A and much higher rates in the two progressors (B and C), with the highest virus evolutionary rate in recipient B in agreement with the linear regression analysis and also consistent with his recent death with AIDS. Thus, from these analyses we have strong evidence showing a considerable influence of viral evolutionary rate on HIV disease progression.

**Table 2:** Parameter estimates and log likelihoods under different clock models

Model	*p*	Log L	Evolutionary rate
Different Rates	34	-24119	n.a.
Global clock	21	-24218	ABC: 2.928 × l0^-^3 (± 0.72 × l0^-^3)
Local clock for A and (BC)	22	-24164	A: 1.308 × l0^-^3 (± 0.19 × 10^-^3), BC: 5.08810^-^3 (± 0.41 × 10^-^3)
Local clock for A, B and C	23	-24156	A: 1.008 × l0^-^3 (± 0.16 × 10^-^3), B: 1.2 × l0^-^2 (± 1.86 × 10^-^3), C: 4.8 × l0^-^3 (± 0.38 × 10^-^3)

^*p *^The amount of parameters used in the model.

^*LogL*^*The log likelihoods*.

## Discussion

In this study we have carried-out detailed analyses of molecular factors that might contribute to HIV disease progression in an epidemiologically-linked cohort in which a HIV-infected non-progressor transmitted virus to recipients who gradually progressed to AIDS. With the help of 15 full-length HIV-1 genomes derived from the cohort members, where time and source of infection were known, we are able to show how various genetic changes following transmission of HIV from a non-progressor (donor A) accompanied disease progression in two recipients (B and C). Previously, Sydney Blood Bank Cohort (SBBC) also identified a similar transmission of HIV-1 from a non-progressor to 5 other recipients, but in this case patients did not progress as they were all infected with a nef-deleted HIV-1 strain [19]. We have investigated host-induced viral divergence, selection pressure, recombination and viral evolutionary rates of HIV-1 strains in this cohort.

It is apparent that following transmission of HIV-1 from the donor A, the 2 recipients B and C gradually deteriorated over a 15-year period to low CD4^+^/CD8^+ ^T cell counts and high viral loads despite the continuation of HAART since 1997. These data suggest a possible role of *in vivo *viral divergence and host selection pressure over time, in the transition of a virus associated with non-progression in the donor, to a virus associated with gradual progression of HIV in the 2 recipients B and C of the cohort. To investigate this, the contribution of recombination to the genetic diversity and consequently disease progression evident in these cohort members was assessed using IST and the Homoplasy test. As our cohort is epidemiologically-linked, classical techniques such as Simplot, which uses a scanning window approach to detect conflicting topologies, are unreliable. Our methods capture conflicting phylogeny signal at the third codon positions and fourfold degenerate sites, which is unlikely to have resulted from selective pressure, thus indicating recombination. For the complete genomes, similar recombination indices were obtained using both tests. Some differences were observed when individual major genes were considered which could be attributed to different methodology and/or different parameters used by the two different algorithms.

Host-imposed immune selection was investigated by scanning dN/dS ratios across the genome. The variation found across the genome was consistent with that found for HIV-1 group M. Of particular interest was the fairly low ratios obtained for the *gag *gene which has been extensively implicated in CTL escape [3,20]. Further investigations of our analysis also indicates which genome regions have high dN/dS ratios. Though various reports have documented the evolutionary constraints placed by overlapping reading frames and secondary structures on RNA viruses such as HIV-1 [21,22], it is important to note that the exact number and location of the identified positively selected sites are not under investigation. Rather this study focuses on attributing the discordant phylogenetic patterns detected over time between cohort members by the possible contribution of positive selection. Differential selective pressure was found to have substantially contributed to virus evolution within these three cohort members.

Furthermore, it is noteworthy that while recombination in addition to selection forces may have contributed to the formation of the virus causing the gradual progression of HIV in the 2 recipients, it is possible that the HIV status of these individuals is associated with their HLA types, and not only due to the possible emergence of CTL escape mutations or other host factors as described previously [7,15,23].

In addition, by investigating the divergence of the serially sampled sequences using linear regression [24], we analyzed the rate of viral evolution. Although this analysis is suggestive of higher evolutionary rates in both progressors, the overlapping confidence intervals do not allow us to conclude significant differences. Earlier reports conducted by Ganeshan *et al*., and Essajee and colleagues based their HIV diversity studies on only partial segments of the *en*v gene [25,26], conducting similar phylogenetic analysis but assessing viral heterogeneity either through heteroduplex assays or nucleotide based distance matrices, respectively. Despite both reports depending only on the *en*v gene, which is naturally variable, both indicate that early quasispecies diversification may be associated with a favorable clinical outcome, with limited heterogeneity correlating to slower HIV disease, and a lack of vertical transmission from mother child pairs, respectively [25,26]. Taken together, literature suggests that an inverse relationship exists between viral diversity and disease progression [25,26], however other studies inclusive of ours also indicate the contrary [15,27]. Moreover, as our analysis relies on predetermined mathematical algorithms the assumption of data independence by linear regression estimates is violated as sequences share a phylogenetic history. Therefore, we estimated the evolutionary rates using a maximum likelihood framework that takes this into account and allows us to test different hypotheses using local clock models imposed onto the genealogy [28,29]. This molecular clock analysis, confirmed a higher rate of evolution in progressors B and C, as opposed to a lower rate in non-progressing donor A. The fact that HIV evolutionary rate could be patient-specific and influenced by immunologic control or even therapy-induced control [30], has major implications for evolutionary and vaccine studies. In our study it is difficult to assess the role of therapy-induced control of HIV-evolution as both patient B and C, who received therapy, had intermittent changes in drug regimen, which usually comprises of a cocktail of drugs and makes it impossible to dissect the role of each drug on the virus. Previous studies have indicated that combinations of RT drugs can act together to further increase HIV-1 mutation frequencies [30]. Thus, although we believe that therapy may have partially influenced viral evolution of HIV-1 strains in cohort patients, it is difficult to assess contribution of individual drugs in affecting viral evolutionary rates. Nonetheless, it is important to reiterate that it does not bias our overall interpretation of HIV disease progression as both recipients prior to initiation of therapy (pre 1997) were showing a gradual decline in T cell counts and rising plasma viremia.

Thus, the most unique aspect of our study the demonstration of patient-specific evolutionary rates as a major contributor to the general lack of a molecular clock in HIV. To date no molecular clock model accommodates for recombination and one can dispute the relevance of the evolutionary rates obtained. However, the genealogy-based estimates are in good agreement with the linear regression estimates, which were based on the viral divergence for each patient separately. Simulations have shown that recombination, even in small amounts, can disturb the molecular clock [31,32], and hence why the more general non-clock model provides a better fit to this data.

Overall, our studies raise the possibility that non-progressors, in some cases may harbor both pathogenic and non-pathogenic variants. Host genetics may act as driving force for positive selection of infecting strains [33]. Although viral recombination and differential selective pressure were found to have significantly affected virus variability in all 3 cohort members, there was striking correlation between faster viral evolutionary rate with accelerated disease progression.

## Materials and methods

### Cohort patient profiles

**Figure 1:**
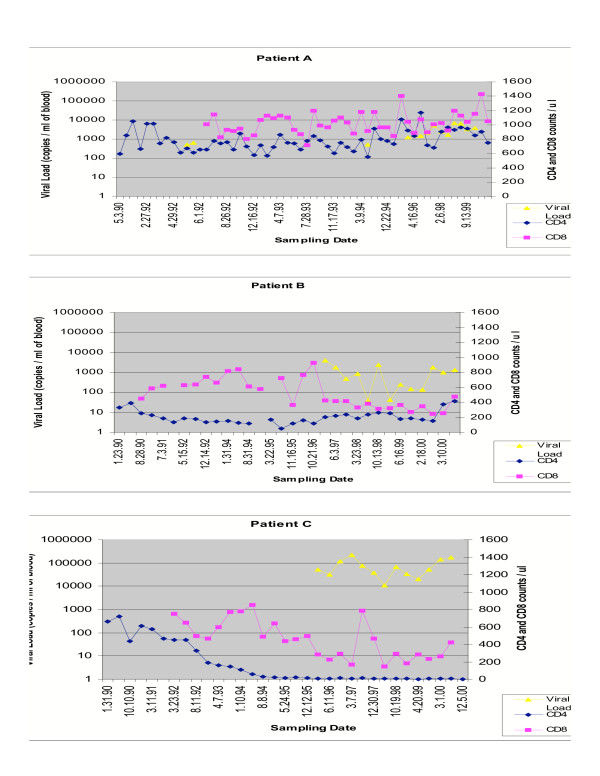
Cohort patient profiles showing CD4+ and CD8+ T cell counts and plasma viral loads for patients A, B and C, respectively.

By using the well-described approaches of both Lookback and Traceback, clusters of distant HIV transmissions can be identified [34]. One such cluster was identified with the donor A, who likely acquired infection in 1982 and infected 2 recipients B (in 1983 autumn) and C (in 1983 summer) through blood transfusion. These infections were confirmed serologically in late 1990. The donor has remained well for over twenty years without requiring antiretroviral therapy and has maintained CD4^+ ^T cell count above 550 cells/mm3 and CD8^+ ^T cell count over 600 cells/mm3 and a viral load consistently less than 10000 copies /ml. In contrast, both recipients (B and C) have required the use of highly active antiretroviral therapy (HAART) which was initiated in 1995 and 1997 respectively (consisting of ddl/3TC/IMD) with recipient B still alive. On the other hand recipient C experienced a dramatic decline in CD4^+ ^T cell count in 1997 down to CD4^+ ^T cell count of 7 cells / mm^3 ^(Figure 1A, IB and 1C) and has recently died of AIDS-related illness after 14 years post-infection. HLA typing was also conducted revealing patient A to be type A2, A3, B57, B65 and unknown for locus C, patient B showed to be HLA A2, A11, B56, B62 and CW1, while patient C was similariy found to be HLA A2, A24, B7, B13 and unknown for locus C. For a detailed description of patient clinical profiles, patient HLA types and phylogenetic evidence confirming epidemiological linkage refer to Mikhail *et al*., 2005.

### Full Length genome amplification of HIV-1 strains

Gene-Amp XL PCR kit (Perkin – Elmer Emerville Ca, USA) together with nested internal PCR reactions were used to amplify near full-length HIV genomes (8766 base pairs, the LTR domains were amplified separately) as previously published [5,15]. Population sequencing was conducted on a total of four longitudinal cohort samples obtained from donor A, termed Al, A3, A5, and A6 and corresponded to years 1992, 1997, 1998 and 2000. Similarity 4 time points from patient B were termed B3, B4, B5 and B6 correspond to years: 1992, 1997, 1998 and 2000 for sample collection, with C2, C3, C5, C6, C8, C10 and C11 representing patient C samples obtained from 1993, 1994, 1996, 1993, 1997, 1998 and 2000. To investigate the presence of patient mutations within a known CTL epitope, a database search was conducted within the Los Alamos (NM, USA) immunology database [18]. HIV-1 near full length sequences derived from cohort patients were consequently used to confirm epidemiological linkage and investigate molecular gene by gene comparisons as previously published [15].

### Sequencing and phylogenetic analysis of cohort patients

Population nucleotide sequences and peptide sequences were aligned using CLUSTAL W [35] and manually edited in Se-AI according to their reading frame. The best-fitting nucleotide-substitution model was selected using Modeltestv3.06 [36], Phylogenetic trees were reconstructed in PAUP4.0bl0, starting from a Neighbor-Joining tree under a heuristic maximum likelihood search that implemented both nearest-neighbor interchange (NNI) and subtree pruning-regrafting (SPR). Bootstrap analysis was performed using the Neighbor-Joining method on 1000 replicates (previously published in Mikhail *et al*., 2005). Bayesian trees were reconstructed in mrBayes v2.01. Network analysis was performed in Splitstree 2.4.

### Recombination analysis

Since the detection of specific recombination patterns and breakpoints in closely related sequences might be unreliable, evidence for recombination was investigated on a non-overlapping DNA concatemer or in single gene regions using two different tests: (a) the Informative Sites Test (IST) as implemented in PIST on the third codon positions [16], and (b) the Homoplasy Test on the fourfold degenerate sites [16]. The Homoplasy Test determines if there is a statistically significant excess of homoplasies in the phylogenetic tree derived from the data set, compared to an estimate of the number of homoplasies expected by repeated mutation in the absence of recombination [37]. An index of greater than zero indicates linkage equilibrium or recombination, but a value of zero or less indicates pure clonal evolution [34], The IST test detects whether the proportion of two-state parsimony-informative sites to all polymorphic sites is greater than expected from clonally generated data [16].

### Selective pressure

Non-synonymous to synonymous substitution rate ratio's (*d*N/*d*S) were estimated in a sliding-window fashion under a probabilistic model of codon substitution that restricts all sites to a single *d*N/*d*S (M0) index across the complete genome [28]. All calculations were performed using the codeml program from the PAML package.

### Evolutionary rate analysis

Root-to-tip divergences were calculated in VirusRates v.0, provided by Andrew Rambaut [24]. Confidence intervals for the linear regression estimates were obtained by bootstrapping the original alignment. Maximum likelihood analysis and local clock modeling was performed in PAML v 3.13 b, provided by Ziheng Yang, which implements a tip-date model estimated as additional parameters under the constraint that the positions of the tips are proportional to the sampling date [28].

## Genbank accession numbers

Near full length HIV-1 genomes derived from cohort patient's PBMCs have been allocated Genebank accession numbers AY779550-AY779564.

## List of abbreviations used

HIV-l human immunodeficiency virus type 1

AIDS acquired immunodeficiency syndrome

PBMC peripheral blood mononuclear cells

IST Informative site test

HR homoplasy ratio

SBBC Sydney blood bank cohort

CTL cytotoxic T lymphocyte

HLA human leukocyte antigen

NNI nearest neighbor interchange

## Competing interests

The author(s) declare that they have no competing interests.

## Authors' contributions

M.M was assisted by B.W in carrying out the molecular genetic studies, generating sequence alignments, and drafting the paper. P.L conducted the evolutionary and recombination studies, B.B together with M.J.G provided the clinical samples, under analysis, while A-M.V participated in the design of the evolutionary study and its analysis. N.K.S conceived of the study, participated in its supervision, design, complete coordination and conclusion. All authors read and approved the final manuscript.
